# Transmitted drug resistance to Tenofovir/Emtricitabine among persons with newly diagnosed HIV infection in Shenyang city, Northeast China from 2016 to 2018

**DOI:** 10.1186/s12879-021-06312-3

**Published:** 2021-07-09

**Authors:** Zhen Wang, Bin Zhao, Minghui An, Wei Song, Xue Dong, Xin Li, Lu Wang, Lin Wang, Wen Tian, Haibo Ding, Xiaoxu Han

**Affiliations:** 1 National Clinical Research Center for Laboratory Medicine, NHC Key Laboratory of AIDS Immunology (China Medical University), The First Affiliated Hospital of China Medical University, No 155, Nanjing North Street, Heping District, Shenyang, 110001 Liaoning Province China; 2 Key Laboratory of AIDS Immunology, Chinese Academy of Medical Sciences, Shenyang, 110001 China; 3 Key Laboratory of AIDS Immunology of Liaoning Province, Shenyang, 110001 China; 4 Department of Food Safety and Nutrition, Shenyang Center for Health Service and Administrative Law Enforcement (Shenyang Center for Disease Control and Prevention), Shenyang, 110031 China

**Keywords:** HIV-1, Pre-exposure prophylaxis, Transmitted drug resistance

## Abstract

**Background:**

To assess transmitted drug resistance (TDR) to tenofovir (TDF)/emtricitabine (FTC), using as pre-exposure prophylaxis, among newly diagnosed human immunodeficiency virus-1 (HIV-1)-infected residents in Shenyang city, northeast China.

**Methods:**

Demographic and epidemiological information of all newly diagnosed HIV-1 infected residents in Shenyang city from 2016 to 2018 were anonymously collected from the local HIV epidemic database. HIV-1 *pol* sequences were amplified from RNA in cryopreserved plasma samples and sequenced directly. Viral subtypes were inferred with phylogenetic analysis and drug resistance mutations (DRMs) were determined according to the Stanford HIVdb algorithm. Recent HIV infection was determined with HIV Limiting Antigen avidity electro immunoassay.

**Results:**

A total of 2176 sequences (92.4%, 2176/2354) were obtained; 70.9% (1536/2167) were CRF01_AE, followed by CRF07_BC (18.0%, 391/2167), subtype B (4.7%, 102/2167), other subtypes (2.6%, 56/2167), and unique recombinant forms (3.8%, 82/2167). The prevalence of TDR was 4.9% (107/2167), among which, only 0.6% (13/2167) was resistance to TDF/FTC. Most of these subjects had CRF01_AE strains (76.9%, 10/13), were unmarried (76.9%, 10/13), infected through homosexual contact (92.3%, 12/13), and over 30 years old (median age: 33). The TDF/FTC DRMs included K65R (8/13), M184I/V (5/13), and Y115F (2/13). Recent HIV infection accounted for only 23.1% (3/13). Most cases were sporadic in the phylogenetic tree, except two CRF01_AE sequences with K65R (Bootstrap value: 99%).

**Conclusions:**

The prevalence of TDR to TDF/FTC is low among newly diagnosed HIV-infected cases in Shenyang, suggesting that TDR may have little impact on the protective effect of the ongoing *CROPrEP* project in Shenyang city.

## Background

Pre-exposure prophylaxis (PrEP) with antiviral drugs in high risk populations is considered an effective way to prevent human immunodeficiency virus (HIV) infection. In 2012, the World Health Organization (WHO) released guidelines to help uninfected people at risk of HIV infection use PrEP [1]. In 2012, Truvada was firstly approved as a PrEP drug by the US Food and Drug Administration (FDA). This compound preparation consists of two nucleoside reverse transcription inhibitors (NRTIs): tenofovir (TDF) and emtricitabine (FTC). Truvada is currently the most widely used PrEP drug in the world [2] and is the only recommended PrEP drug in US Centers for Disease Control guidelines [3]. In 2015, WHO guidelines recommended PrEP by oral administration of antiviral drugs containing TDF (TDF + FTC, or TDF + lamivudine [3TC], or TDF) [4]. In 2019, Descovy was the second PrEP drug approved by the US FDA, and its active ingredients included FTC and tenofovir alafenamide (TAF, TDF prodrug) [5]. PrEP strategies in high-risk populations have been approved by many countries and regions including the United States, Canada, the European Union, Australia, Kenya, and South Africa [3, 6]. However, no PrEP drug has been approved in China yet.

As early as 2010, TDF was recommended as the first-line drug of antiretroviral therapy (ART) for acquired immunodeficiency syndrome (AIDS) by the US and European AIDS clinical societies [7]. FTC is also the first-line drug of ART recommended by the WHO. The main drug resistance mutations (DRMs) to TDF/FTC include K65R and M184I/V, but the global incidence was rather low in HIV ART-naïve patients [8]. The Stanford drug resistance database showed the incidence of K65R (1.6–3.0%) and M184I/V (30–63%) among eight common HIV-1 subtypes in NRTI-treated patients [8]. Therefore, the breakthrough infections caused by HIV with DRMs from ART-failure patients may compromise the effect of PrEP [9]. In a recent report on HIV-infected MSM in four US cities, the rate of drug resistance was as high as 30, and 16% were resistant to PrEP drugs [10]. Different from the relatively high number of DRMs in developed countries, the latest meta-analysis in China showed that although acquired drug resistance (ADR, develops because of viral replication in the presence of antiretroviral drugs [11]) for NRTI among ART-treated patients reached 31.4%, the transmitted drug resistance (TDR, detected among ARV drug-naive people with no history of antiretroviral drug exposure [11]) for NRTI among HIV ART-naïve patients was as low as 0.7% [12]. There have been many reports about HIV TDR in China, but none have focused on DRMs related to PrEP.

Shenyang city is the capital of Liaoning Province, the center city of Northeast China. In recent years, men who have sex with men (MSM) have become the population most affected by HIV infection in Shenyang, where diverse HIV strains have been reported for 20 years [13, 14], and there are extensive connections with strains among MSM throughout China [15, 16]. The TDR rate among MSM in Shenyang was reported as 4.5–4.7% before 2015 [13, 17]. MSM represents a key HIV high-risk population, which accounts for more than a quarter of all new infections in China [18] and over three-quarters of new HIV infections in Shenyang. At the end of 2018, *CROPrEP*, a multicenter, real-world prospective cohort study aiming at a comprehensive evaluation of the PrEP feasibility in China, was started among MSM in four metropolitan Chinese cities with moderate to high HIV prevalence rates among MSM, including Shenyang [19]. To explore the baseline TDRs in Shenyang city, especially the TDRs that compromise the effect of TDF/FTC before the *CROPrEP* project started, we retrospectively analyzed the TDR of all newly diagnosed HIV-infected cases in Shenyang between 2016 and 2018.

## Methods

### Study subjects

This study was a retrospective molecular epidemiology study. All newly diagnosed HIV-infected residents of Shenyang between 2016 and 2018 were included in this study. Cryopreserved plasma samples at diagnosis of HIV infection were available in 2354 cases (91.3%, out of 2577). Demographic information including gender, age, marital status, education level, and HIV infection route was also collected anonymously. The Institutional Review Board of China Medical University approved this study.

### HIV *pol* gene amplification and sequencing

Briefly, HIV-1 RNA was extracted from 140-μl plasma samples using QIAamp Viral RNA Mini kit (Qiagen, Hilden, Germany), and the HIV *pol* region gene (HXB_2:_ 2253–3318) was reverse transcribed and amplified with an in-house method. Polymerase chain reaction (PCR) products were purified and then directly sequenced in both directions. Amplification primers, PCR conditions, and sequencing primers were described previously [13].

### Sequences assembly, phylogenetic, and TDR analyses

The *pol* sequences were aligned using Vector NTI Advance 10.0 Software Contig Express Component (Invitrogen, Carlsbad, CA, USA). HIV-1 subtype reference sequences were downloaded from the Los Alamos database (https://www.hiv.lanl.gov). A Maximum-Likelihood (ML) tree (GTR nucleotide substitution model) for CRF01_AE was constructed using fast tree 2.1.8 [20]. A bootstrap value > 90 was the criterion to determine lineage [15]. FigTree1.4.3 (http://tree.bio.ed.ac.uk/software/figtree) was used for phylogenetic analyses.

The assembled sequences were submitted to the Stanford University HIV Drug Resistance Database (http://hivdb.stanford.edu) to identify the DRMs. Only those DRMs that could cause low, moderate, and high-level resistance to antiretroviral drugs were recorded and counted.

### HIV-1 limiting antigen avidity detection

HIV-1 Limiting Antigen Avidity (LAg-Avidity) kits (Maxim Biomedical, Inc., USA) were used to distinguish recent HIV infection (RHI) from chronic HIV infection (CHI). The tests were performed according to the kit instructions [21]. The normalized optical density (ODn) of each sample = optical density (OD) value of each sample/OD value of the calibrator. If ODn > 2.0 in the screening test, it is judged as CHI. If ODn ≤2.0, a confirmatory test is required. ODn > 1.5 in a confirmatory test indicates CHI, while ≤1.5, is considered RHI [21].

### Statistical analyses

All patients with PREP-related resistance mutations will be included in the analysis. Statistics of all demographic data including gender, age, marital status, education level, and HIV infection route, and statistics of TDR were performed using SPSS version 25.0 (SPSS Inc., Chicago, IL). We analyzed continuous variables and categorical variables. Descriptive statistics were conducted and related factors were summarized and sorted.

## Results

### Demographic characteristics

A total of 2167 (92.1%, 2167/2354) sequences were acquired. The overall distribution of subtypes was: 70.9% CRF01_AE (1536/2167), 18.0% CRF07_BC (391/2167), 4.7% subtype B (102/2167), 2.6% other subtypes (56/2167), and 3.8% unique recombinant forms (82/2167). Among the cases whose subtype could be determined, 93.9% (2034/2167) were male with a median age of 34 years old (range: 4–90), 63.1% (1364/2167) were unmarried, 49.6% (1075/2167) had a college education or above, and 83.6% (1811/2167) were MSM.

### Total TDRs and resistance to TDF/FTC

Overall, 4.9% of sequences in this study (107/2167) were found to harbor DRMs. The DRMs to protease inhibitors (PIs), NRTIs, and nonnucleoside reverse transcriptase inhibitors (NNRTIs) were 1.0% (21/2167), 0.5% (10/2167), and 2.8% (61/2167), respectively. Among them, 0.6% (13/2167) of sequences were resistant to TDF/FTC, including 10 of CRF01_AE, 2 of CRF07_BC, and 1 subtype B strains. The mutations included K65R (61.5%, 8/13), M184I/V (38.5%, 5/13), and Y115F (2/13). K65R is known to confer high-level resistance to TDF and intermediate-level resistance to FTC, while M184I/V confers high-level resistance to FTC and 3TC and low-level resistance to abacavir (ABC). Y115F can confer high-level resistance to ABC and only low-level resistance to TDF (Table 1).

**Table 1:** Information of HIV-1 infected persons with TDF/FTC-related TDR in Shenyang in 2016–2018

NO.	Year of detection	Subtype	Mutation	Drug Resistance^**a**^		Infection stage^**b**^
				TDF	FTC	
1	2017	CRF01_AE	K65R	H	I	CHI
2	2018	CRF01_AE	K65R	H	I	CHI
3	2016	CRF01_AE	K65R	H	I	NA
4	2017	CRF01_AE	K65R	H	I	CHI
5	2018	CRF01_AE	K65R	H	I	NA
6	2016	CRF01_AE	K65KR	H	I	RHI
7	2016	CRF01_AE	M184I	S	H	NA
8	2016	CRF01_AE	M184M/V	S	H	CHI
9	2017	CRF01_AE	M184M/V	S	H	RHI
10	2018	CRF01_AE	Y115F, M184V	S	H	NA
11	2018	CRF07_BC	K65K/R	H	I	RHI
12	2017	CRF07_BC	K65K/R	H	I	CHI
13	2017	B	K70K/E,Y115Y/F,M184V	I	H	CHI

^a^*H* high-level resistance, *I* intermediate-level resistance, *S* susceptible

^b^*RHI* Recently HIV Infection, *CHI* Chronic HIV Infection, *NA* Not Available

Among the patients resistant to TDF/FTC, 92.3% (12/13) were male with a median age of 33 years old (range: 25–50), 76.9% (10/13) were unmarried, 46.1% (6/13) had a college education or above, 76.9% (10/13) were MSM, and 23.1% (3/13) were estimated to having been infected within 6 months.

### Phylogenetic relationships of TDF/FTC-resistant strains

**Fig. 1:**
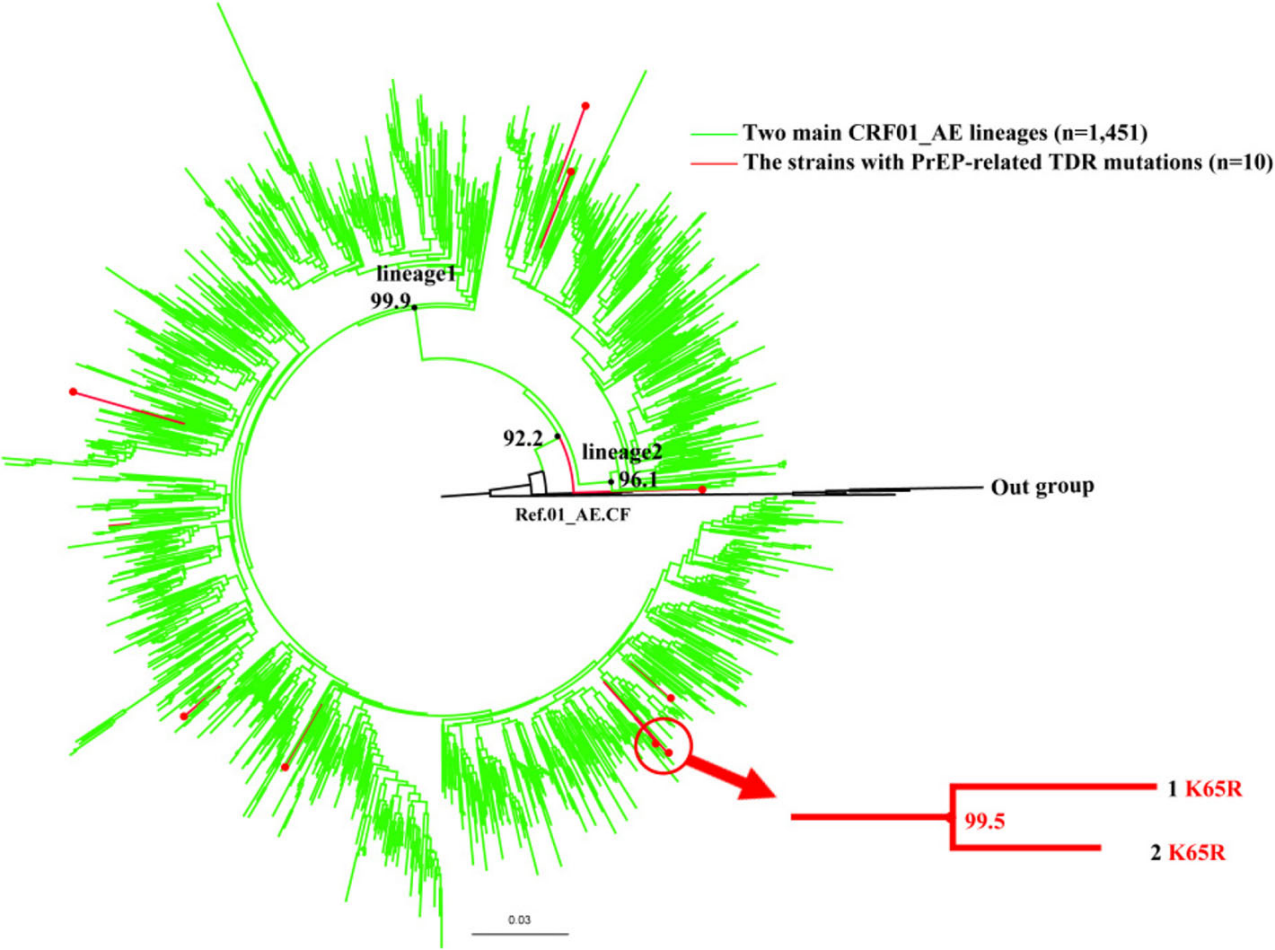
The maximum-likelihood tree was constructed based on the *pol* region (HXB2: 2253 to 3300 nt). HIV-1 subtype B was chosen as out-group in the CRF01_AE rooted tree. Only the sequences located in 2 main CRF01_AE lineages (95.1%, 1461/1536) were included in this ML tree. The strains with TDF/FTC-related PDR mutations (red) located in 2 main lineages of CRF01_AE (green)

A majority (76.9%, 10/13) of TDF/FTC resistant strains in this study were classified as CRF01_AE, so linkage among the TDF/FTC resistant strains was phylogenetically analyzed against the other TDF/FTC sensitive CRF01_AE strains. In the ML tree, 95.1% (1461/1536) of sequences could be classified as two main CRF01_AE lineages that were described in a previous study on the Chinese MSM population [15]. Eight TDF/FTC resistant strains were sporadic in the ML tree without obvious aggregation, but the other two CRF01_AE strains with K65R clustered together with 99% of the bootstrap value (Fig. 1). There was no significant correlation between the two CRF07_BC strains and K65K/R (data not shown).

## Discussion

ART for HIV-infected patients has been effective in reducing the mortality of HIV infection and preventing an ongoing HIV epidemic in the absence of vaccines [22]. China’s Free ART Program, initiated in 2002 [23], developed from an emergency response to a standardized treatment. At present, 17 ART drugs are provided free by the Chinese government, including 4 NRTIs (zidovudine [AZT], lamivudine [3TC], TDF and abacavir [ABC]), 2 NNRTIs (efavirenz [EFV] and nevirapine [NVP]), and 1 PI (lopinavir/r [LPV/r]). Stavudine (d4T)/AZT + 3TC + NVP/ EFV was the earliest, free, first-line ART regimen in China in 2005 [24]. After that, TDF-based ART regimens (combination TDF + 3TC + EFV) were used according to the 2011 guideline of diagnosis and treatment for AIDS in China [25]. By October 30, 2019, there were 829,628 HIV-infected patients receiving ART, accounting for 86.6% of HIV-1 infections in China [26]. The virological suppression rate exceeded 90% among those on ART [27], but drug resistance inevitably occurs with wide ART application. The situation of HIV-1 drug resistance in China from 2001 to 2017 has been comprehensively evaluated [12]. The prevalence rates of TDR and ADR in China were about 3.0 and 44.7%, respectively, depending on the regions studied. It is no surprise that NNRTI-related mutations increased rapidly in China recently, and M184V/I and T215I/Y/S/D/F were the main NRTI DRMs in ART-naïve and ART-treated individuals, respectively [12].

In this study, we explored TDR to TDF/FTC among all newly diagnosed HIV infections in Shenyang city from 2016 to 2018. We found three DRMs (K65R, M184I/V, and Y115Y/F) that caused resistance to TDF/FTC. K65R and M184I/V were both listed in the common DRMs to TDR and ADR [12] and were believed to be induced under the pressure of TDF and 3TC [28]. Both were widely used in first-line ART regimens [29] and confer high-level resistance to TDF and FTC. Y115Y/F was not a common DRM and could be selected by ABC and TDF [28], both of which were free ART drugs in China and confer high-level resistance to ABC and low-level resistance to TDF. Therefore, TDR to TDF/FTC found in this study was most likely related to the previous application of first-line ART drugs in China.

In this study, TDR to TDF/FTC was detected in 13 out of 2176 patients. Are there any similarities or links among these patients? Our results showed that most were unmarried, infected HIV through homosexual behavior and that three had likely been infected within 6 months. Phylogenetic analysis indicated that most TDR strain transmission was sporadic, except for two K65R carrying CRF01_AE strains with very close genetic distance. These findings suggest that although there is a potential risk of TDR strain local transmission, that risk is still low.

The most important question arising from TDR transmission is whether PrEP will protect the individuals who are exposed to HIV strains with DRMs against TDF/FTC. Animal models have shown that oral TDF/FTC did not reliably prevent infection with HIV strains with K65R [30]. In humans, studies have shown that some individuals acquired HIV strains with DRMs against TDF and/or FTC despite consistent use of adequate doses of PrEP [9, 31]. Therefore, PrEP does not offer complete protection against HIV infection, especially HIV strains with DRMs. The results of this study showed that the prevalence rate of TDR to TDF/FTC was as low as 0.6% among newly diagnosed HIV-infected cases in Shenyang, suggesting that TDR may have little impact on the protective effect of the ongoing CROPrEP project in Shenyang city [19]. At last, the objects of our study was not exactly the same as the target population of the *CROPrEP* project in Shenyang city, and included other HIV high-risk population. This could be the limitation of this study.

## Conclusion

In summary, this is the first comprehensive large-scale local investigation on the transmission of TDR strains to TDF/FTC in China. The low prevalence and few phylogenetic link of HIV TDR in Shenyang suggested that the impact of TDR on the ongoing PrEP project is very limited. However, the overall prevalence of HIV TDR was close to 5% in Shenyang, highlighting the need for regular TDR monitoring to prevent the further spread of HIV TDR strains.

## Data Availability

The datasets used and/or analyzed during the current study are available from the corresponding author upon reasonable request.

## Abbreviations

HIV: Human immunodeficiency virus
PrEP: Pre-Exposure Prophylaxis
TDR: Transmitted Drug Resistance
MSM: Men who have sex with men
RHI: Recent HIV infections
